# Comparative Evaluation of Esthetic Zirconia Crowns in Children: A Comprehensive Review

**DOI:** 10.7759/cureus.93704

**Published:** 2025-10-02

**Authors:** Ameera M Alrassan, Sama S Alsaad, Hawraa F Alsunni, Sarah H Alsalman, Fahad K Almasoudi, Maha I Alsane, Khallad Z Alqarni, Mona A Masmali, Mazen A Alhodaithy, Daniah A Basheer, Tala S Khider, Moodhi N Alsubaei, Bushra M Wasili, Amal H Alsaffar

**Affiliations:** 1 College of Dentistry, King Saud Bin Abdulaziz University for Health Sciences, Riyadh, SAU; 2 Pediatric Dentistry, King Saud Medical City, Ministry of Health, Riyadh, SAU; 3 Dentistry, Ministry of Health, Al-Ahsa, SAU; 4 College of Dentistry, King Khalid University, Abha, SAU; 5 Dentistry, Ministry of Health, Jazan, SAU; 6 Dentistry, Ministry of Health, Qassim, SAU; 7 Dentistry, Ministry of Health, Tabuk, SAU; 8 Faculty of Dentistry, King Abdulaziz University, Jeddah, SAU; 9 Dentistry, Ministry of Health, Abha, SAU; 10 Pediatric Dentistry, Ministry of Health, Qatif, SAU

**Keywords:** children, esthetic crowns, pediatric dentistry, primary teeth, zirconia crowns

## Abstract

Restoration of primary teeth is a fundamental component of pediatric dental care. While traditional crown types have long been used, the introduction of zirconia crowns has marked a significant advancement in terms of esthetics, durability, and biological compatibility. Given their increasing popularity, a narrative evaluation of zirconia crowns relative to other available options is warranted. Therefore, this comprehensive narrative review aims to provide an evidence-based comparison of zirconia crowns with other restorations used for primary teeth. By synthesizing data from current literature, this review aims to offer clinicians a detailed understanding of the comparative advantages, limitations, and clinical considerations associated with zirconia crowns in pediatric dental rehabilitation. Zirconia crowns provide a clinically superior option for rehabilitation of primary teeth, combining esthetic excellence with functional durability. Their advantages across multiple outcome domains support their increasing adoption in pediatric dentistry. Continued research into long-term outcomes will further optimize their clinical utility.

## Introduction and background

Carious lesions can rapidly progress in primary teeth due to their anatomical characteristics, thin enamel, large pulp chambers, and broad contact points, necessitating full-coverage restorations. Historically, stainless steel crowns (SSCs) have been the mainstay for restoring extensively damaged primary teeth due to their durability, ease of use, and cost-effectiveness [1]. However, with growing societal emphasis on esthetics, particularly among parents, the demand for more esthetically acceptable restorative alternatives has surged. In response to the limitations of SSCs, prefabricated zirconia crowns have been introduced and rapidly adopted in pediatric dental practice. These all-ceramic crowns offer several advantages, including superior esthetics, biocompatibility, and resistance to plaque accumulation, making them a desirable alternative to metal-based restorations [2]. Introduced commercially in the late 2000s, zirconia crowns are now available for primary teeth, with various manufacturers offering design variations to enhance fit, retention, and clinical handling [3].

Zirconia's intrinsic mechanical properties, such as high flexural strength and fracture toughness, combined with its inert, polished surface, offer both structural resilience and reduced bacterial adhesion [4]. However, unlike SSCs, zirconia crowns require more aggressive tooth reduction and cannot be crimped or adapted intraorally, presenting both clinical challenges and biological considerations, especially in younger patients with limited cooperation [5]. Over the past decade, multiple clinical trials, observational studies, and laboratory investigations have evaluated zirconia crowns in comparison to other full-coverage restorations, including SSCs, strip crowns, composite resin-based crowns, and preveneered metal crowns. These studies have examined a wide range of outcomes, from periodontal response and crown retention to esthetic satisfaction and long-term durability. Yet, despite a growing body of literature, a comprehensive synthesis that systematically compares zirconia crowns to all other alternatives across all relevant clinical domains remains limited.

Although prefabricated zirconia crowns are widely adopted, clinical training opportunities remain uneven, and accessibility differs depending on region and manufacturer. Additionally, comparative evidence on their clinical performance remains fragmented. Given the broadening application of zirconia in pediatric dentistry, a critical evaluation of its effectiveness is essential. Pediatric dentists, especially those operating in esthetically driven practices, need evidence-based guidance to inform crown selection based not only on appearance but also on functional outcomes. Therefore, this comprehensive narrative review aims to provide an evidence-based comparison of zirconia crowns to other full-coverage restorations used for primary teeth. By synthesizing data from current literature, this review aims to offer clinicians a detailed understanding of the comparative advantages, limitations, and clinical considerations associated with zirconia crowns in pediatric dental rehabilitation.

## Review

Search strategy

**Figure 1:**
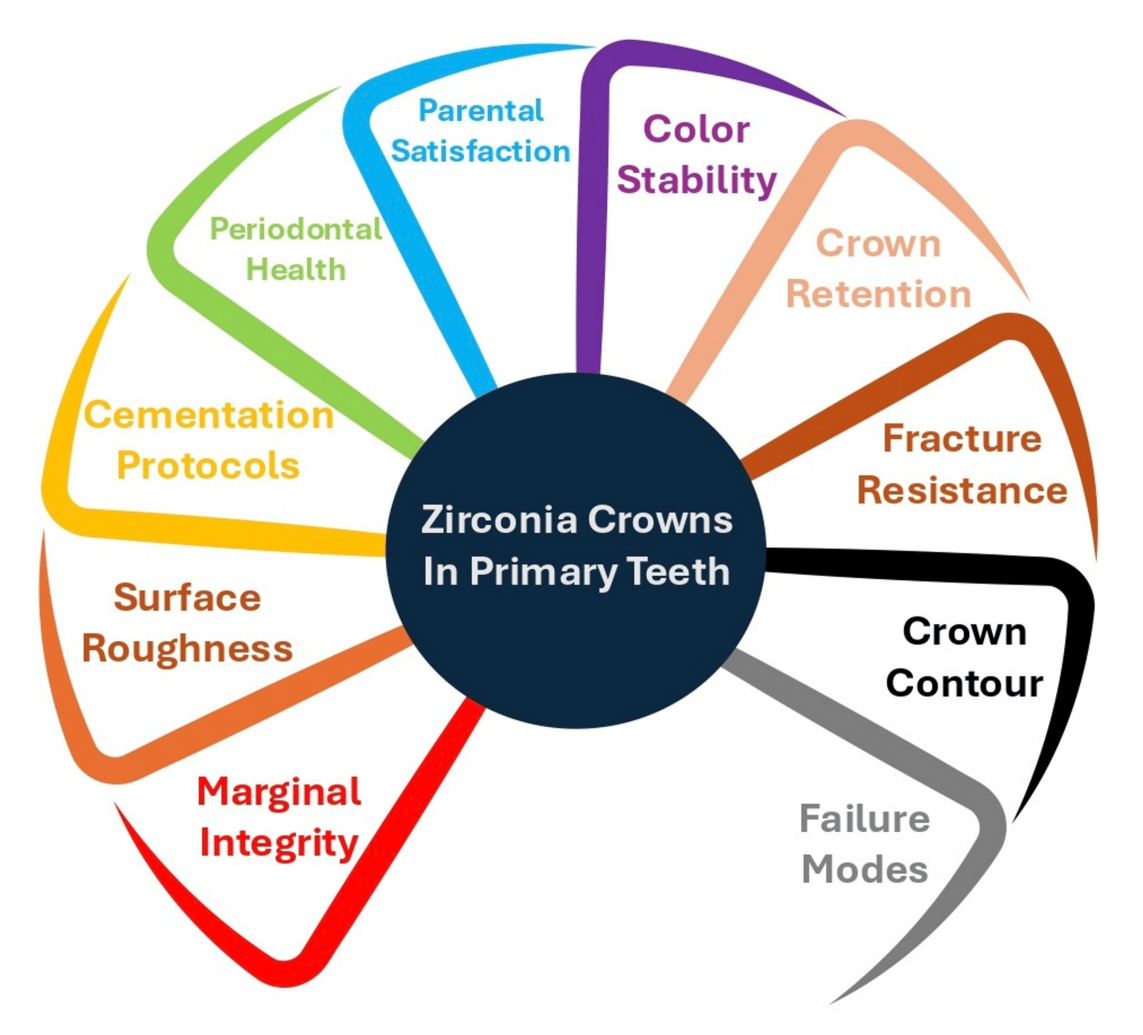
Overview of domains evaluated Created by K.Z.A. with the help of Canva

A literature search was conducted across major scientific databases, including PubMed, Scopus, and Web of Science. The search focused on articles published in English, with emphasis on those examining the effectiveness of zirconia crowns in pediatric dentistry. Specific keywords and Boolean combinations were used, such as “zirconia crowns,” “pediatric dentistry,” “primary teeth,” “stainless steel crowns,” “strip crowns,” and “preformed crowns,” Outcome-related search terms were also included, covering the clinical domains subsequently evaluated in this review: periodontal health, parental satisfaction, color stability, crown retention, crown contour and anatomy, fracture resistance, marginal integrity, surface roughness, cementation materials, and failure modes and longevity. Selection was guided by relevance to the predefined outcome domains evaluated in this review. References cited in eligible articles were also explored to identify additional pertinent studies. The selected literature formed the scientific foundation for the thematic synthesis presented throughout this review. A range of clinical domains was evaluated to comprehensively compare the performance of zirconia crowns with alternative restorations. These domains are summarized in Figure 1.

Periodontal health

The preservation of gingival health is central to the long-term success of full-coverage restorations in pediatric patients [6]. Evidence consistently shows that zirconia crowns provide more favorable periodontal outcomes compared to SSCs and other restorative options. In both clinical trials and observational studies, zirconia crowns have been associated with lower plaque accumulation and reduced gingival inflammation, findings attributed to their highly polished and non-porous surfaces, which discourage bacterial adhesion [7,8]. By contrast, SSCs are more prone to plaque retention due to surface irregularities and less favorable cervical adaptation. The cervical margins of SSCs, in particular, often act as plaque-retentive niches, predisposing children with limited oral hygiene to gingivitis [9]. Preveneered SSCs and composite strip crowns perform less favorably still, with reports of bulkier margins, rougher surfaces, and veneer fractures or delamination. These factors increase the risk of localized soft tissue inflammation and compromise the gingival seal [10,11]. The biologic advantages of zirconia crowns are not only material-dependent but also design-related. Prefabricated zirconia crowns exhibit rounded anatomical contours that mimic natural dentition, promoting self-cleansing and reducing food impaction [12]. Clinical assessments further support their favorable gingival response, with fewer cases of bleeding on probing and soft tissue swelling compared to SSCs [7,13]. In addition, zirconia’s chemical stability and absence of ion release eliminate the risk of localized inflammatory reactions that can occur with metal-based restorations [13]. Taken together, the literature suggests that zirconia crowns may demonstrate superior periodontal compatibility relative to SSCs, preveneered SSCs, and strip crowns. While the durability of SSCs is well established, their plaque-retentive nature places them at a biological disadvantage. Zirconia crowns, in contrast, combine esthetics with improved gingival health, making them particularly advantageous for both anterior and posterior restorations where tissue preservation is critical.

Parental satisfaction

**Figure 2:**
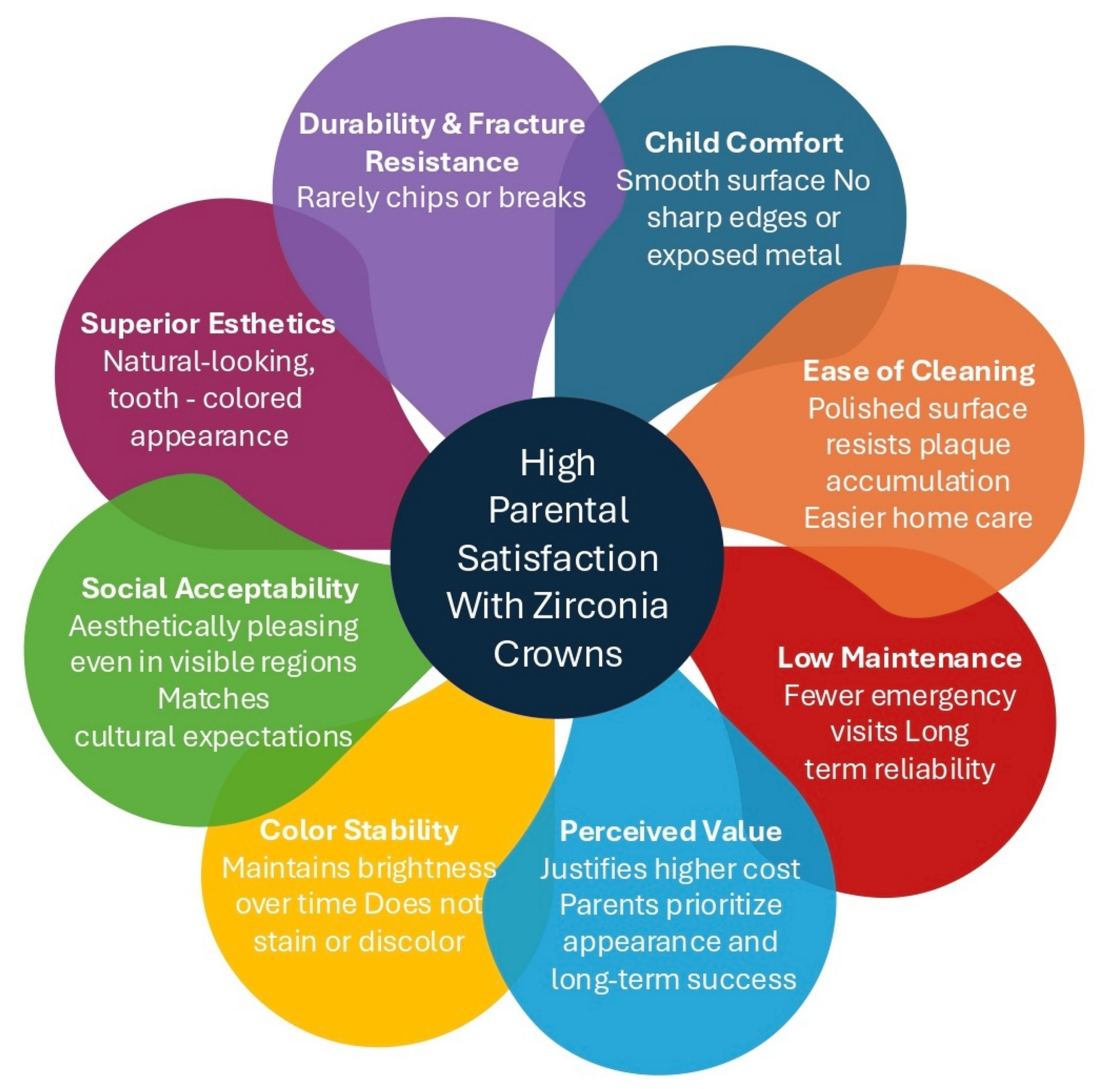
Core elements underlying high parental satisfaction with zirconia crowns Created by K.Z.A. with the help of Canva

Parental acceptance is an important determinant of treatment success in pediatric restorative dentistry. Evidence consistently shows that zirconia crowns outperform SSCs in esthetic domains, which strongly influences caregiver satisfaction. Several randomized controlled trials have demonstrated that parents prefer zirconia crowns due to their natural tooth-like color and shape, with satisfaction ratings significantly higher than those reported for SSCs [14-16]. This preference is particularly pronounced for anterior teeth, where esthetics are of primary concern. Nevertheless, the literature also highlights nuances. Some studies found that while esthetic satisfaction was markedly superior with zirconia crowns, functional outcomes such as retention and durability were valued more by caregivers of children restored with SSCs [15,17]. In certain cases, parents expressed equal satisfaction when SSCs provided long-term stability and avoided repeat treatment [18]. Moreover, cultural and socioeconomic factors appear to influence these perceptions; in populations where treatment cost is a limiting factor, parental preference may not always align with the higher esthetic expectations associated with zirconia crowns [16]. Comparisons with alternative esthetic restorations further reinforce zirconia’s advantage. Composite strip crowns, though initially well-received esthetically, often show discoloration or fracture over time, leading to decreased parental satisfaction at follow-up [19,20]. Preveneered SSCs similarly suffer from veneer chipping and bulkier contours, which compromise both appearance and gingival health, resulting in mixed parental feedback [14]. By contrast, zirconia crowns maintain esthetic stability throughout the follow-up periods reported, sustaining high levels of satisfaction [15,18]. Overall, the literature suggests that zirconia crowns provide superior esthetic outcomes that directly translate into higher parental satisfaction when compared to SSCs, preveneered SSCs, and strip crowns. However, this advantage must be weighed against considerations of cost, tooth preparation requirements, and clinical longevity, which may influence parental perceptions differently across treatment contexts (Figure 2).

Color stability

The long-term success of esthetic full-coverage restorations in children depends not only on retention but also on color stability. Zirconia crowns generally demonstrate superior resistance to discoloration when compared to composite strip crowns and preveneered SSCs. Their densely sintered microstructure and glazed surface reduce surface porosity, thereby limiting staining from dietary sources and minimizing plaque accumulation that could alter crown appearance [21]. Clinical follow-up studies confirm that zirconia crowns maintain a stable shade over time, even in cases with extended observation periods [22]. In contrast, composite strip crowns are highly susceptible to staining and discoloration, particularly in children with frequent exposure to colored beverages. Several studies have reported noticeable changes in shade within 12 to 24 months of placement, a factor that contributes to reduced parental satisfaction at follow-up [22,23]. Similarly, preveneered SSCs demonstrate compromised color stability due to veneer fractures, delamination, or discoloration at the veneer-metal interface, leading to esthetic deterioration over time [21]. Nevertheless, it is important to note that zirconia crowns are not entirely immune to color change. Experimental work has demonstrated that exposure to acidic beverages and pigmented solutions can result in minor but measurable alterations in shade, though the magnitude of these changes is generally below the threshold of clinical perceptibility [24]. Such findings suggest that while zirconia crowns outperform other restorative options, their long-term esthetic performance may still depend on patient-specific dietary and oral hygiene factors. Unlike composite resin-based restorations or strip crowns, which are susceptible to staining and shade mismatch over time, zirconia crowns do not undergo chemical degradation or absorb pigments [22]. Their shade-matched, factory-finished surfaces are designed to remain color-stable even in children with high caries risk or poor oral hygiene, making them an ideal choice for visible teeth. Comparatively, preveneered SSCs, which incorporate resin-based facings, often exhibit color instability due to surface wear, chipping, and loss of glaze [23]. These changes can expose underlying metal or lead to visible shade differences between the veneered surface and the surrounding tooth structure. Similarly, composite strip crowns may initially provide good esthetics, but their long-term color performance is often compromised by microcracks, surface wear, and absorption of dietary chromogens [25]. The superior optical properties of zirconia, including its light-reflective and translucent characteristics, also enhance its visual integration with natural teeth. This optical compatibility contributes to a more lifelike appearance, particularly in anterior restorations, and ensures that the crown remains esthetically acceptable even as surrounding teeth change slightly in color due to growth or environmental factors [26]. From a practical standpoint, the color stability of zirconia crowns reduces the need for future re-intervention or replacement due to esthetic deterioration [27]. This is particularly valuable in pediatric patients, where repeated dental procedures can be distressing and may undermine cooperation. The ability to place a crown that maintains its esthetic integrity until natural exfoliation offers a major advantage in clinical efficiency and patient experience. Zirconia crowns provide outstanding color stability, outperforming all other common pediatric crown types in terms of long-term esthetic maintenance [28]. Their resistance to staining, combined with durable glazing and lifelike translucency, ensures that they remain visually acceptable and natural-looking. Taken together, the comparative evidence indicates that zirconia crowns exhibit the most stable color outcomes among pediatric full-coverage restorations. While strip crowns and veneered SSCs often degrade in esthetic quality over time, zirconia crowns retain their visual appeal, strengthening their role as the restoration of choice when esthetics are prioritized.

Crown retention

Retention is a fundamental requirement for the long-term success of full-coverage restorations in pediatric dentistry [29]. A crown that fails to remain in place undermines both function and esthetics and may necessitate additional clinical appointments, which can be distressing for young patients and caregivers. In this context, the retention capacity of different crown types, particularly zirconia crowns versus other alternatives, is an essential area of comparison. Zirconia crowns, while inherently rigid and non-retentive by mechanical means such as crimping or flexing, achieve secure retention primarily through passive fit and strong adhesive bonding with appropriate luting agents [30]. These crowns are prefabricated with standardized internal dimensions and rely on accurate tooth preparation to provide a snug, friction-based fit. The inner surfaces of many zirconia crowns are micro-mechanically enhanced, via surface grooves, sandblasting, or texturing, to improve adhesion with resin-modified glass ionomer or other adhesive cements [31]. When placed with proper technique, zirconia crowns exhibit excellent clinical retention. Their performance is largely dependent on the precision of tooth preparation, including uniform circumferential reduction and the absence of undercuts. Because zirconia crowns cannot be crimped or adjusted chairside, the preparatory phase becomes critical to ensure a passive, fully-seated fit. Once cemented, the crowns tend to remain stable throughout the exfoliative life of the primary tooth [32]. In comparison, SSCs are highly retentive due to their ability to be crimped and adapted around the tooth structure [33]. This mechanical advantage allows SSCs to compensate for less-than-ideal preparations and contributes to their reputation as the most retentive crown type overall. However, their retention is mechanical rather than adhesive, and in cases of marginal leakage or cement washout, they may still loosen or require recementation [34]. Preveneered SSCs and strip crowns generally have inferior retention characteristics compared to both zirconia and traditional SSCs. Preveneered crowns may lose their facing due to resin fracture or debonding, especially under occlusal stress [35]. Strip crowns, which rely on resin composites bonded to the tooth structure, are particularly prone to loss due to wear, marginal breakdown, or failure of the bonding interface, especially in moist environments or when oral hygiene is compromised [36]. While zirconia crowns require more aggressive tooth reduction than other types, this reduction contributes to increased surface area for bonding and helps in achieving long-term retention. When combined with proper isolation, careful cementation, and postoperative instructions, the risk of dislodgement is low. Moreover, the need for recementation or repair is rare, offering advantages in clinical efficiency and patient compliance [37]. Zirconia crowns provide reliable and predictable retention when placed using correct clinical protocols. Although they lack the mechanical adaptability of metal crowns, their combination of passive fit and strong adhesive bonding ensures excellent stability. Their retention performance, combined with esthetics and durability, makes zirconia crowns a dependable option for full-coverage restoration in primary teeth [38].

Crown contour and anatomy

The anatomical contour of pediatric crowns is central to occlusal function, esthetics, and gingival compatibility. Zirconia crowns, produced using CAD/CAM technology, are prefabricated with standardized anatomy that closely mimics natural primary teeth, including defined cuspal slopes and smooth interproximal surfaces [39,40]. Data suggest that these features support efficient occlusal force distribution and reduce plaque retention compared with less anatomically precise crowns [40]. By contrast, SSCs, though highly durable, often require crimping and adaptation during placement. These adjustments can flatten occlusal surfaces and distort interproximal form, leading to less accurate contacts and potential areas for plaque accumulation [41,42]. Preveneered SSCs present further limitations, as the addition of a veneer layer frequently results in bulkier contours that compromise emergence profile and gingival adaptation [43]. Composite strip crowns, while capable of esthetic outcomes at placement, depend heavily on operator technique to achieve proper contour. Over time, wear and discoloration can degrade their anatomy and compromise both function and appearance [43]. A key advantage of zirconia crowns is their consistency. Unlike chairside-fabricated strip crowns, their prefabricated design ensures uniform morphology across cases, reducing operator variability and contributing to predictable outcomes [44]. This anatomical realism not only enhances esthetics but also supports periodontal health by minimizing mechanical irritation and food impaction, features less reliably achieved with SSCs or strip crowns [40,42]. Taken together, comparative evidence indicates that zirconia crowns provide superior anatomical accuracy and contour compared with SSCs, preveneered SSCs, and strip crowns. Their consistent morphology supports favorable occlusion and esthetics while promoting soft tissue health, making them the most anatomically precise option among available pediatric full-coverage restorations.

Fracture resistance

Fracture resistance is a key determinant of clinical longevity for pediatric full-coverage restorations. Zirconia crowns consistently demonstrate higher fracture loads compared to strip crowns and preveneered SSCs, especially in children affected by molar-incisor hypomineralization, where weakened enamel significantly increases the risk of structural failure [45]. Laboratory testing has shown that zirconia crowns withstand significantly greater occlusal forces before failure, a property attributed to their monolithic structure and high flexural strength [46]. These findings indicate that zirconia crowns are particularly suited for posterior applications where masticatory forces are greatest. This strength arises from its dense crystalline structure, which resists crack propagation and deformation under stress. Even in thin sections, it can absorb masticatory forces effectively, minimizing the risk of catastrophic failure [47]. This mechanical advantage is particularly important in posterior teeth, where occlusal forces are greatest, and in children with bruxism or other high-stress conditions. In clinical practice, zirconia crowns exhibit minimal incidences of fracture or chipping once properly placed. Unlike preveneered SSCs, which are prone to delamination of their esthetic facings, zirconia crowns are monolithic and do not have veneering layers that can separate or fracture [48]. By contrast, composite strip crowns and resin-based restorations tend to be more susceptible to fracture and wear over time. Their relatively lower mechanical properties and higher susceptibility to moisture or thermal stress make them less durable, particularly in posterior applications [49]. Preveneered SSCs, while mechanically supported by the metal substructure, often fail esthetically due to fracture of the veneer, which compromises both appearance and functionality. SSCs themselves are highly fracture-resistant but lack the esthetic qualities that many parents and patients desire. Another advantage of zirconia crowns is their ability to maintain fracture resistance over time. They are highly resistant to degradation from saliva, temperature fluctuations, or pH changes, all of which can weaken other restorative materials. Their toughness also allows them to withstand accidental trauma, a common occurrence in pediatric populations, without chipping or cracking [13]. From a clinical perspective, the high fracture resistance of zirconia crowns translates to fewer emergency visits and a lower need for reintervention, which is particularly valuable in young patients who may be anxious or uncooperative. This durability also reduces long-term treatment costs and improves parental satisfaction by ensuring the restoration remains intact until natural exfoliation of the primary tooth. Zirconia crowns demonstrate superior fracture resistance compared to other pediatric full-coverage restorations. Their monolithic construction, robust mechanical properties, and resistance to oral environmental challenges make them an excellent choice for both anterior and posterior teeth, where reliability and longevity are paramount [50].

Marginal integrity

The marginal adaptation of pediatric crowns is a critical factor for protecting tooth structure, reducing microleakage, and maintaining gingival health. Crowns with poor marginal fit can promote plaque accumulation, recurrent caries, or gingival irritation. Zirconia crowns are manufactured with computer-aided design and milling, producing standardized internal dimensions and smooth, well-defined margins. In vitro investigations have shown that finish line design significantly affects their marginal accuracy, with chamfer and shoulder preparations providing more favorable adaptation than vertical finish lines [51]. In clinical practice, the marginal adaptation of zirconia crowns is highly technique-sensitive. Because zirconia does not permit chairside crimping or contouring, success depends entirely on precise preparation and seating. Undercuts, irregular margins, or insufficient axial reduction can result in incomplete seating or open margins. When prepared and cemented properly, however, zirconia crowns form a thin and continuous luting interface that resists bacterial infiltration and promotes long-term stability [51]. Compared to other options, SSCs offer greater adaptability due to their ductile nature. Their margins can be crimped and burnished to closely follow the cervical contour of the prepared tooth, which can be particularly beneficial when ideal preparation is difficult to achieve. However, this adjustability can also introduce variability. Over-crimping or improper adaptation may lead to ledging, marginal overhangs, or subgingival extension, all of which can irritate the gingiva or create plaque-retentive zones [52]. Preveneered SSCs, while similar in structure to traditional SSCs, often feature a bulkier facial surface due to the added resin layer. This additional thickness may hinder optimal marginal adaptation, especially on the labial side, where over-contouring and difficulty in achieving a flush cervical seal are more common. Furthermore, once the veneering resin begins to chip or wear, the exposed interfaces may trap debris and compromise the seal [53]. Strip crowns and composite restorations are highly dependent on bonding and adaptation at the time of placement. While a good marginal seal can initially be achieved, degradation over time due to resin wear, shrinkage, or microleakage is a known concern. Their performance in terms of marginal integrity is also highly sensitive to technique factors, including moisture control, matrix adaptation, and polymerization dynamics [54]. One of the benefits of zirconia is its polished cervical margin, which facilitates easy cement removal and creates a smooth transition from crown to tooth. This reduces the likelihood of residual cement acting as an irritant or leading to plaque buildup. In addition, the rigidity of zirconia ensures that marginal gaps are less likely to widen under functional loading or over time [55]. Zirconia crowns provide excellent marginal integrity when the preparation and cementation protocols are executed correctly. While they lack the intraoral adaptability of metal crowns, their structural precision and material stability offer a consistent and biologically compatible marginal seal [56]. This advantage is most clearly realized in cases where meticulous technique can be ensured, and it contributes significantly to the overall success and longevity of the restoration.

Surface roughness

Surface roughness is an important determinant of plaque retention, bacterial adhesion, and gingival health. Smooth crown surfaces facilitate oral hygiene and esthetic stability, which are particularly critical in pediatric patients who may not consistently maintain optimal oral hygiene. Zirconia crowns are distinguished by their polished, glazed surfaces that provide superior smoothness compared with other crown types. Laboratory studies confirm that polishing and glazing markedly reduce surface roughness and mitigate the risk of excessive enamel wear on opposing teeth [57]. Their non-porous structure contributes to resistance against discoloration and surface degradation. Clinical observations further emphasize that zirconia crowns provide a surface feel similar to natural enamel, with margins that are both comfortable and gingivally compatible [58]. SSCs, in contrast, often display micro-roughness following crimping or burnishing. These irregularities can create plaque-retentive niches and increase the likelihood of gingival irritation over time [59]. Preveneered SSCs initially improve esthetics, but long-term studies demonstrate deterioration of the resin veneer, including chipping and surface defects that compromise smoothness [60]. Composite strip crowns, though capable of achieving a polished surface at placement, are particularly prone to degradation; wear from brushing and dietary factors leads to increased roughness and diminished gloss retention [61]. The implications of surface roughness extend to antagonist tooth wear. When zirconia is properly polished and glazed, it does not accelerate enamel wear compared with other ceramic systems [62]. Conversely, roughened surfaces on stainless steel or composite restorations can contribute to attrition, especially in children with bruxism. Importantly, clinical case reports highlight that the smooth, natural esthetics of zirconia restorations also enhance patient acceptance and satisfaction, particularly in the anterior region where appearance and comfort are highly valued [63]. In summary, zirconia crowns demonstrate superior surface smoothness, resistance to degradation, and favorable biological response compared with stainless steel, preveneered, or composite strip crowns. These features promote plaque control, esthetic longevity, and patient comfort, underscoring zirconia as a preferred option for pediatric full-coverage restorations.

Cementation materials

The choice and performance of cementation materials are vital for the clinical success of pediatric crowns. It is worth mentioning that a durable bond between the crown and tooth structure ensures retention, seals the margins to prevent microleakage, and contributes to long-term restoration stability. The interaction between the crown material and the luting agent also influences the risk of postoperative sensitivity, crown dislodgement, and recurrent decay [64]. Zirconia crowns differ from other pediatric crown types in their cementation requirements. Due to their rigid, non-retentive structure and lack of crimpability, zirconia crowns rely heavily on adhesive bonding to achieve reliable retention [65]. This necessitates careful selection and handling of appropriate luting agents. Resin-modified glass ionomer cements are among the most commonly used options due to their combination of adequate bond strength, fluoride release, ease of use, and moisture tolerance, which are key advantages in pediatric dentistry where absolute dryness is often difficult to maintain. Alternatively, some clinicians use self-adhesive resin cements to achieve higher bond strengths, particularly in cases where maximum retention is needed. These cements provide excellent mechanical properties and bonding performance but are more technique-sensitive and may require stricter isolation or surface conditioning of the tooth. The inner surface of many prefabricated zirconia crowns is also pretreated or roughened to enhance micromechanical retention with adhesive cements. A successful cementation protocol with zirconia crowns involves thorough tooth preparation, proper fit verification, moisture control, and complete seating with firm pressure [66]. Once set, excess cement must be meticulously removed to prevent subgingival irritation or cement-induced inflammation. The smooth surface of zirconia facilitates easy cement cleanup, especially at the margins, reducing the risk of postoperative complications. SSCs, in contrast, typically depend less on adhesive bonding and more on mechanical retention through crimping and adaptation. Conventional glass ionomer cements are frequently used with SSCs, offering sufficient retention and fluoride release. These cements are user-friendly, cost-effective, and forgiving in wet environments, making them well-suited for use with stainless steel restorations. However, their lower bond strength compared to resin-based cements makes them less ideal for materials like zirconia, which require strong adhesive support [67]. Preveneered SSCs and composite strip crowns involve even more complexity. The former still relies on mechanical retention but may present challenges during cementation due to bulkier contours or irregular surfaces. The latter requires etching, bonding, and layering of composite, with increased sensitivity to contamination and technique errors. Strip crowns must also achieve a strong chemical bond with the tooth, and any compromise in the bonding protocol can lead to early failure [68]. Ultimately, the effectiveness of the crown-tooth interface hinges on the compatibility of the luting agent with the crown material. Zirconia crowns demand modern adhesive techniques and proper case selection to achieve optimal outcomes. Although they may be slightly more demanding in terms of cementation technique compared to SSCs, their long-term stability and reduced need for retreatment often justify the initial effort [69]. Zirconia crowns require adhesive-based cementation protocols, typically using resin-modified glass ionomer or self-adhesive resin cements. These materials provide a reliable seal and strong retention when applied with proper technique. The success of zirconia restorations depends on understanding and applying these principles, which differ significantly from the mechanical approaches used with metal-based crowns.

Failure modes and longevity

The long-term success of full-coverage restorations in primary teeth is contingent not only on their initial esthetic and functional performance but also on their ability to withstand clinical stresses and biological challenges over time. Patterns of failure vary among crown types and provide valuable insights into material selection, technique optimization, and patient-centered treatment planning. Prefabricated zirconia crowns have demonstrated encouraging survival outcomes in both short- and medium-term studies. Cohort data indicate survival rates exceeding 90% at two years, with reports of favorable outcomes extending to more than six years when adhesive protocols are followed meticulously [70]. Clinical failures are relatively uncommon and typically involve loss of retention due to cement failure, rather than catastrophic fracture of the crown itself. Importantly, pulpal status appears to influence longevity: crowns placed on pulpotomized teeth exhibit slightly higher failure rates compared with those on vital teeth, underscoring the need for cautious case selection. Overall, zirconia’s monolithic structure minimizes fracture-related complications, but its dependence on precise preparation and adhesive cementation makes debonding the most frequent cause of failure. SSCs remain the most durable option from a purely mechanical standpoint, with decades of clinical evidence confirming their longevity. Failures most often involve cement loss and marginal leakage, which may predispose to recurrent caries. In contrast to zirconia, mechanical crimping allows SSCs to compensate for less-than-ideal tooth preparation, but this adaptability also introduces variability in marginal integrity. Although survival rates are high, esthetic concerns frequently prompt parental dissatisfaction, leading to elective replacement despite clinical functionality. Bioflx crowns have recently been introduced as an esthetic and potentially cost-effective alternative. Randomized controlled trials suggest that their clinical performance is broadly comparable to zirconia and SSCs at one to two years, though evidence remains limited [69]. Failures in this group are primarily linked to debonding, reflecting the same cementation sensitivity observed with zirconia [71]. Long-term data beyond the two-year mark are lacking, which precludes definitive conclusions about their survival relative to more established options. Composite strip crowns and preveneered SSCs remain vulnerable to esthetic deterioration over time. Veneer chipping, color instability, and wear are the predominant failure modes, often resulting in early replacement. Although these restorations may initially satisfy esthetic demands, their reduced durability limits their usefulness in cases where long-term stability is required [29]. Zirconia and SSCs consistently outperform other crown types in terms of longevity, though their failure modes differ. Zirconia crowns are highly resistant to fracture but require meticulous technique to avoid debonding. SSCs, while mechanically resilient, are more prone to marginal leakage and esthetic rejection. Bioflx crowns offer a promising alternative, yet evidence remains preliminary. Methodological limitations across studies-including small sample sizes, heterogeneity in outcome measures, and relatively short follow-up durations-make it difficult to draw definitive conclusions about long-term survival. Standardized reporting of failure modes and extended follow-up are necessary to generate more robust comparative data.

Clinical implications

**Figure 3:**
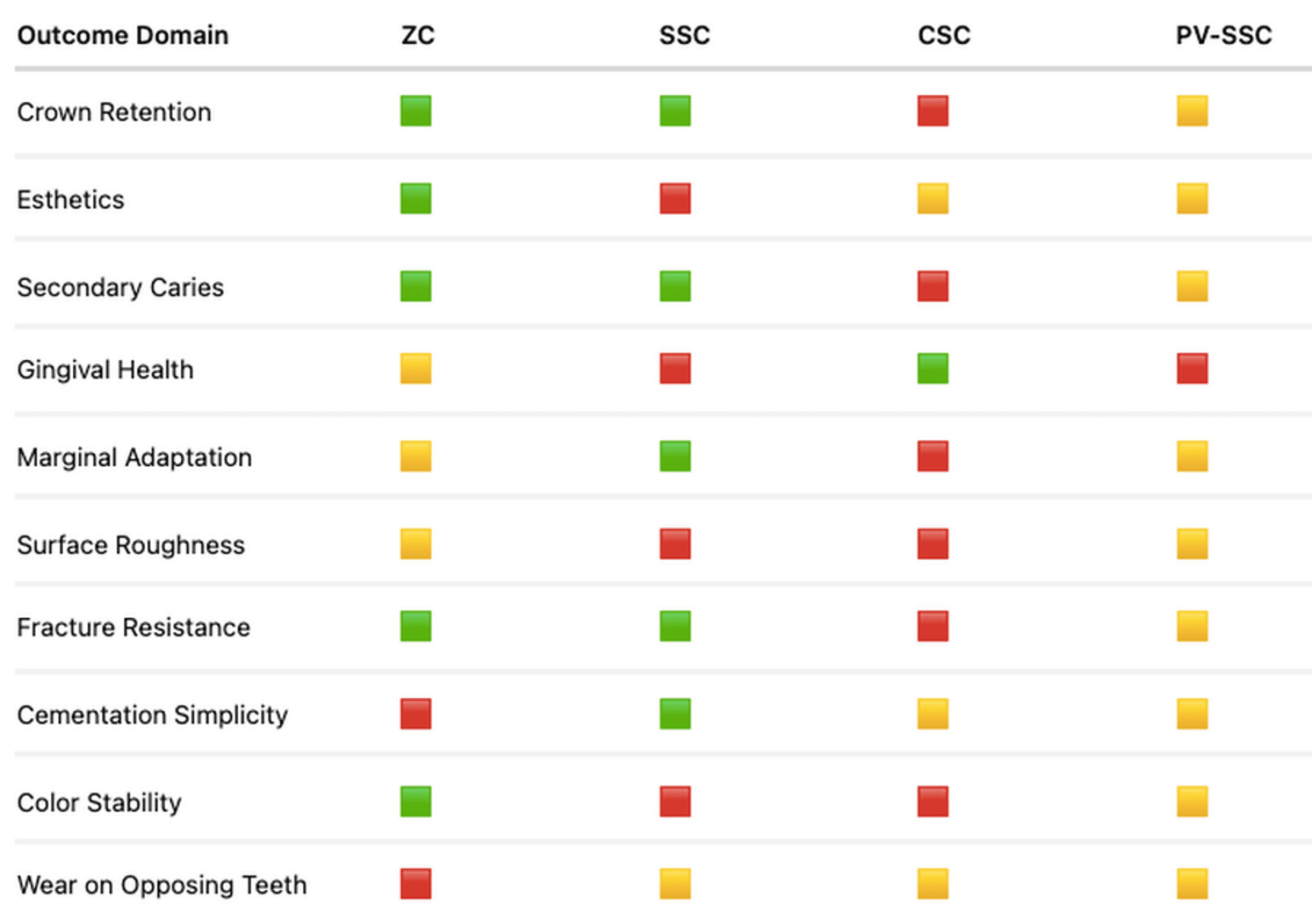
Comparative clinical profile of zirconia and other pediatric crown types Green indicates high performance, yellow indicates moderate performance, and red indicates low performance. ZC, zirconia crown; SSC, stainless steel crown; CSC, composite strip crown; PV-SSC, preveneered stainless steel crown.

The increasing adoption of zirconia crowns in pediatric dentistry reflects a broader shift toward more esthetic, biologically compatible, and durable restorative options for children. Understanding the clinical implications of using them allows practitioners to integrate them effectively into daily practice and make informed decisions depending on patient needs and expectations. A comparative summary of clinical attributes across different pediatric crown types is presented in Figure 3.

Case Selection and Patient Considerations

Zirconia crowns are especially advantageous in cases where esthetics is a priority, such as in anterior restorations or in children whose parents express concern about the appearance of traditional metal crowns. They are also ideal for patients with a history of allergy to nickel or other metals, as zirconia is biocompatible and non-allergenic. Additionally, children with high caries risk, poor oral hygiene, or limited access to regular dental care benefit from zirconia’s durability and caries-preventive features. However, these crowns may not be suitable for every case. Patients with extremely small teeth, crowding, or limited eruption may present challenges during placement due to the need for aggressive reduction and passive fit. Younger children with large pulp chambers or uncooperative behavior may also require additional caution due to the risk of pulp exposure and the demand for precision [72].

Clinical Technique and Training

The use of zirconia crowns requires strict adherence to specific preparation guidelines. Unlike SSCs, they cannot be adjusted once selected, making accurate reduction and crown selection critical. This elevates the importance of training and familiarity with zirconia systems, including understanding size selection, preparation design, and cementation protocols. Clinicians must also be equipped to manage isolation and moisture control during cementation, especially when using adhesive systems. Mastery of the technique not only enhances clinical success but also reduces chair time, which is vital in pediatric patients [73].

Practice Efficiency and Workflow

Despite a steeper learning curve, zirconia crowns can improve efficiency in the long term. Their durability reduces the need for follow-up visits, repairs, or emergency care, which benefits both clinicians and families. The prefabricated nature of zirconia crowns also shortens the operative time once the technique is mastered, as no finishing or polishing is needed post-placement. In multidisciplinary settings, such as hospital dentistry or under general anesthesia, zirconia crowns offer a dependable solution with minimal risk of future complications-making them particularly useful in treating medically compromised or behaviorally challenging patients in a single visit [74].

Parent Communication and Acceptance

Parents usually seek restorations that preserve their child’s appearance, particularly in social or school settings. Zirconia crowns provide an opportunity for clinicians to meet these expectations without compromising function. When explained properly, their esthetic and hygienic advantages often outweigh concerns about cost. Clear communication about the preparation needs, durability, and long-term benefits is essential for building trust and ensuring informed consent [75].

Access-to-Care 

While the upfront cost of zirconia crowns may be higher, their longevity, reduced maintenance needs, and superior esthetic outcomes may justify their use in selected cases within public health settings. Expanding insurance coverage and inclusion in treatment planning protocols could further support their wider use in underserved communities. The most frequently cited benefits of zirconia crowns include esthetic superiority and favorable periodontal response, whereas their technique sensitivity and cost remain limitations (Table 1).

**Table 1:** Advantages and limitations of zirconia crowns

Category	Advantages	Limitations
Esthetics	- Highly esthetic tooth-like appearance - Superior color stability over time - No visible metal or veneering components	- Limited shade options - Cannot be customized chairside - May not match adjacent dentition in all cases
Biocompatibility	- Excellent tissue response - Smooth, polished surfaces reduce plaque adhesion - Hypoallergenic and metal-free	- No fluoride release
Durability	- High fracture resistance - Withstands occlusal load in posterior teeth - Long-term clinical success documented	- No intraoral crimping or adjustment possible
Retention	- Excellent passive fit when proper tooth reduction is achieved - High bonding strength with proper cementation	- Technique-sensitive bonding protocol - Requires aggressive and precise tooth reduction to ensure fit
Periodontal health	- Smooth margins promote better gingival adaptation - Less inflammation and bleeding compared to SSCs	- Subgingival margins may still cause transient irritation if overextended
Caries prevention	- Less prone to marginal leakage - Reduces risk of recurrent caries if margins are intact	- No inherent fluoride release unlike some other materials
Patient acceptance	- High acceptance due to esthetics - Parents often prefer zirconia over metallic crowns in visible zones	- Younger children may not tolerate longer procedure times or require general anesthesia due to complexity
Tooth preparation	- Predefined sizing simplifies crown selection - No finishing/polishing after cementation	- Requires significant circumferential and occlusal reduction - Higher risk of pulp exposure in young teeth with large pulp chambers
Cementation	- Compatible with strong adhesive systems - Excellent marginal seal with resin-modified glass ionomer or self-adhesive resin cements	- Sensitive to moisture contamination during bonding
Time efficiency	- Once mastered, quick placement with minimal need for adjustment	- Steeper learning curve for clinicians
Crown adjustability	- Preformed shapes save time in some cases	- Cannot be trimmed, crimped, or reshaped intraorally - Inaccurate fit requires crown replacement
Cost and accessibility	- Fewer failures and esthetic complaints	- Higher material cost

Limitations

Despite the promising outcomes associated with zirconia crowns, the literature reveals several ongoing controversies and limitations. One key debate concerns the extent of tooth reduction required for proper placement. While SSCs can be crimped and adapted with minimal preparation, zirconia crowns demand aggressive reduction to achieve a passive fit, raising concerns about pulp exposure and long-term vitality of primary teeth in younger patients.

Another unresolved issue is the impact of zirconia’s hardness on opposing enamel. Although polished zirconia demonstrates low abrasiveness, in vitro studies suggest potential for enamel wear in bruxism cases, but long-term in vivo data in children remain scarce. The cost-benefit balance is another area of discussion. While parents often prefer zirconia crowns for esthetic reasons, their higher cost may limit accessibility, particularly in public health or low-resource settings. Finally, the lack of intraoral adjustability is a major drawback; once a zirconia crown fails to seat properly, replacement with another crown is necessary, which increases chairside time and cost. These limitations underline the importance of critical case selection and further clinical research to clarify long-term outcomes.

Future research directions

While zirconia crowns have gained widespread acceptance in pediatric dentistry, several important areas remain underexplored. Most clinical evidence to date has emphasized anterior teeth, leaving the long-term performance of zirconia crowns in posterior regions less thoroughly evaluated. Future studies should investigate survival rates, fracture resistance, and marginal adaptation of zirconia crowns used in molars, especially under the influence of higher occlusal forces. Additionally, research is needed to assess how these crowns perform across diverse pediatric populations, considering differences in dietary habits, oral hygiene behaviors, socioeconomic status, and underlying health conditions. Large-scale, multicenter studies with longer follow-up periods could help determine the generalizability of outcomes and identify risk factors for failure. Crown design and fit remain another area ripe for innovation. Current prefabricated systems may not always align with variations in arch form or tooth morphology, especially in mixed dentition. Future research could focus on developing digitally fabricated or semi-custom zirconia crowns that offer improved anatomical fit and better adaptability. Advances in digital workflows, including intraoral scanning and chairside milling, could further enhance customization and efficiency in clinical settings. In parallel, investigations into zirconia surface modifications, such as bioactive coatings or antimicrobial treatments, may improve biological outcomes by reducing plaque accumulation and caries risk. The potential impact of zirconia’s hardness on opposing enamel also warrants further study. While zirconia is generally considered safe when polished, long-term data evaluating enamel wear, especially in children with parafunctional habits, are limited. Research comparing various luting agents is also needed to establish optimal cementation protocols under real-world pediatric conditions. This includes assessing the performance of resin-modified glass ionomer, self-adhesive resin, and bioactive cements in terms of bond strength, ease of use, and clinical longevity. Finally, comprehensive cost-effectiveness analyses and longitudinal assessments of parental satisfaction are essential to support broader adoption. While zirconia crowns may have higher initial costs, their durability and reduced need for retreatment may offer economic advantages over time. Understanding caregiver perceptions of esthetics, durability, and maintenance burden will help clinicians better tailor treatment recommendations and improve communication. Collectively, these future research directions will ensure that the clinical use of zirconia crowns continues to evolve with innovation, precision, and patient-centered care.

Public health perspective

The clinical acceptance of zirconia crowns has grown significantly in high-income countries, largely driven by parental esthetic demands and greater insurance coverage [76,77]. However, in low-and middle-income countries, SSCs remain the predominant choice due to cost-effectiveness, ease of placement, and durability [78]. The disparity raises important public health considerations: should zirconia crowns be prioritized in all cases, or reserved for select situations where esthetics or allergy concerns outweigh financial and technical constraints?

As manufacturing costs decrease and digital workflows become more widespread, zirconia crowns may become more accessible globally. However, integration into public dental programs will depend on demonstrating cost-effectiveness, including reduced retreatments and improved oral health-related quality of life. Cultural factors also play a role; in societies where dental appearance strongly influences social acceptance, parents may be more willing to bear higher costs for zirconia crowns. Addressing these disparities requires both economic analyses and policy-driven approaches to ensure equitable access to esthetic and durable restorative options for children.

## Conclusions

Zirconia crowns have become an important option in pediatric restorative dentistry, providing an esthetic, durable, and biocompatible alternative to traditional crown types. Yet, their use requires a thorough understanding of preparation protocols, cementation techniques, and case selection. The lack of intraoral adjustability, greater tooth reduction demands, and higher material costs must be carefully considered during treatment planning. Despite these challenges, when executed properly, zirconia crowns offer unmatched reliability and esthetic outcomes in pediatric patients.
